# Retrospective Clinical Trial to Evaluate the Effectiveness of a New Tanner–Whitehouse-Based Bone Age Assessment Algorithm Trained with a Deep Neural Network System

**DOI:** 10.3390/diagnostics15080993

**Published:** 2025-04-14

**Authors:** Meesun Lee, Young-Hun Choi, Seul-Bi Lee, Jae-Won Choi, Seunghyun Lee, Jae-Yeon Hwang, Jung-Eun Cheon, SungHyuk Hong, Jeonghoon Kim, Yeon-Jin Cho

**Affiliations:** 1 Department of Radiology, Seoul National University Hospital, 101 Daehak-ro, Jongno-gu, Seoul 03080, Republic of Korea; 2 Department of Radiology, Seoul National University College of Medicine, Jongno-gu, Seoul 03080, Republic of Korea; 3 Institute of Radiation Medicine, Seoul National University Medical Research Center, Jongno-gu, Seoul 03080, Republic of Korea; 4 Healthhub Co., Ltd., AI Lab., Seoul 04175, Republic of Koreakimjh@healthhub.kr (J.K.)

**Keywords:** artificial intelligence, deep learning, bone age measurement, radiography

## Abstract

**Background/Objectives**: To develop an automated deep learning-based bone age prediction model using the Tanner–Whitehouse (TW3) method and evaluate its feasibility by comparing its performance with that of pediatric radiologists. **Methods**: The hand and wrist radiographs of 560 Korean children and adolescents (280 female, 280 male, mean age 9.43 ± 2.92 years) were evaluated using the TW3-based model and three pediatric radiologists. Images with bony destruction, congenital anomalies, or non-diagnostic quality were excluded. A commercialized AI solution built upon the Rotated Single Shot MultiBox Detector (SSD) and EfficientNet-B0 was used. Bone age measurements from the model and radiologists were compared using the paired *t*-tests. Linear regression analysis was performed and the coefficient of determination (r²), mean absolute error (MAE), and root mean square error (RMSE) were measured. A Bland–Altman analysis was conducted and the proportion of bone age predictions within 0.6 years of the radiologists’ assessments was calculated. **Results**: The TW3-based model demonstrated no significant differences between bone age measurements and radiologists, except for participants <6 and >13 years old (overall, *p* = 0.874; 6–8 years, *p* = 0.737; 8–9 years, *p* = 0.093; 9–10 years, *p* = 0.301; 10–11 years, *p* = 0.584; 11–13 years, *p* = 0.976; <6 or >13 years, *p* < 0.001). There was a strong linear correlation between the model prediction and radiologist assessments (r^2^ = 0.977). The RMSE and MAE values of the model were 0.529 (95% CI, 0.482–0.575) and 0.388 (95% CI, 0.361–0.417) years. Overall, 82.3% of bone age model predictions were within 0.6 years of the radiologists’ interpretation. **Conclusions**: Automated deep learning-based bone age assessment has the potential to reduce radiologists’ workload and provide standardized measurements for clinical decision making.

## 1. Introduction

Bone age, typically assessed by radiographic evaluation of the left hand and wrist, is an indicator of skeletal maturity. Clinically, bone age is useful for evaluating the delay and acceleration of puberty, and is well correlated with other biological growth markers [1,2]; notably, the diagnosis of endocrine disorders, including hypothyroidism, growth hormone deficiency, and hypogonadism, all of which may induce changes in bone maturation [1,3]. Children and adolescents undergoing orthopedic surgeries involving growth plates also require precise measurements of bone age to predict and minimize limb-length discrepancies [4].

Among the several methods used for bone age evaluation, the Greulich–Pyle (GP) method is the most commonly used [5]. It involves comparing a radiograph to approximately 30 standard images showing representative cases of bone maturation from the age of 0–18 or 19 years for the corresponding sex. Then, bone age is determined by finding the standard image with the closest match. The simplicity of this method enables fast interpretation, measuring approximately 1.4 min per radiograph [6,7]. However, it suffers from considerable intraobserver (0.18–0.85 years) and interobserver (0.00–3.17 years) variability owing to its dependence on subjective evaluation [8].

In contrast, the Tanner–Whitehouse (TW) method is a score-based system that provides a more detailed assessment by evaluating the maturity level of individual bones [9]. The most recent TW3 method was refined from TW1 by limiting the bones being evaluated from the initial 20 to the radius, ulna, metacarpal bones, and phalanx [1]. As the structural characteristics of different bones are considered, the TW method yields a more accurate and less variable estimation than the GP method [10,11]. However, because the scores of all regions of interest (ROIs) must be added to obtain the final bone age, the TW3 method requires more radiologist effort and a longer interpretation time (approximately 7.9 min) [11,12]. The complexity of the TW method is a major disadvantage, limiting its use in clinical practice.

Recently, artificial intelligence (AI) has opened new avenues for managing complex medical imaging tasks such as the detection and classification of lesions, segmentation of anatomical structures, and registration [13]. In particular, convolutional neural network (CNN)-based deep learning networks have been widely exploited in various fields, such as the detection and classification of thoracic lesions, automated liver segmentation, breast cancer screening, and cancer risk prediction [14,15,16,17].

Several AI solutions have been developed and commercialized for bone age assessment, and previous literature has shown a strong correlation between AI predictions and reference bone ages, along with faster reading time and reduced variability [18,19,20,21,22]. A recent systematic review by Dallora et al. summarized that recent machine learning-based bone age assessment systems automate evaluation with a mean average error (MAE) of 9.96 months [23]. When TW3-model-based AI architectures are applied, the error is reduced to 6 months [24,25,26]. However, these AI models face several limitations. Many of these systems have been developed for and validated in Caucasian populations, raising concerns about their performance in different ethnic groups where skeletal maturation patterns can differ. Moreover, GP-based models have limited resolution in bone age assessment regardless of the size or quality of the training dataset due to their reliance on atlas-based interpretation. Furthermore, current models exhibit unreliable performance with poor-quality images, which may limit their clinical applicability in real-world settings. Therefore, there is a need for robust AI models that can overcome these challenges and be validated across non-Western populations.

In this study, we aimed to evaluate the performance of a TW3-based deep learning model for fully automated bone age assessment. The model employed an architecture combining Rotated Single Shot MultiBox Detector (SSD) and EfficientNet-B0 for ROI extraction and maturity evaluation. We compared the bone ages estimated by the model with those measured by expert pediatric radiologists, using a Korean population in a real-world clinical setting.

## 2. Materials and Methods

### 2.1. Selection of Study Participants

We collected radiographs of the hands and wrists of patients aged ≤15 years, captured at Seoul National University Children’s Hospital between 1 January 2020 and 30 April 2021. A random sample of 2000 radiographs (1000 males and 1000 females) was initially selected using a picture archiving and communication system. The exclusion criteria were as follows: (1) destruction or deformities of the hand and wrist from traumatic, infectious, inflammatory, or metabolic diseases; (2) congenital anomalies of the hand and wrist; and (3) non-diagnostic image quality. After applying the exclusion criteria, we performed age group and sex-stratified random selection to include a total of 560 subjects, with 40 males and 40 females in each age group (<6, 6–8, 8–9, 9–10, 10–11, 11–13, and ≥13 years). The sample size for each group was estimated through power analysis based on an equivalence test for the difference of two means using a paired *t*-test, with the significance level at 0.05, power at 0.9, standard deviation at 0.8, and equivalence margin at 0.6.

### 2.2. Bone Age Assessment by Radiologists

Three board-certified pediatric radiologists (Y.H.C., Y.J.C., and S.B.L., with 20, 11, and 9 years of experience in radiology, respectively) evaluated bone age from all 560 radiographs using the TW3 method, which measures the maturity of the radius, ulna, and 11 short bones in fingers 1, 3, and 5 [9]. Each radiograph was reviewed twice by two radiologists (Y.H.C. and Y.J.C.), with four assessments per image. A 3-week interval was maintained between the interpretations of each radiologist to avoid recall bias. A third radiologist (S.B.L.) determined the final bone age by consensus if at least three out of four readings agreed. If fewer than three readings agreed, the third radiologist selected the most appropriate bone age from among the four readings.

### 2.3. Bone Age Assessment by AI

**Figure A1:**
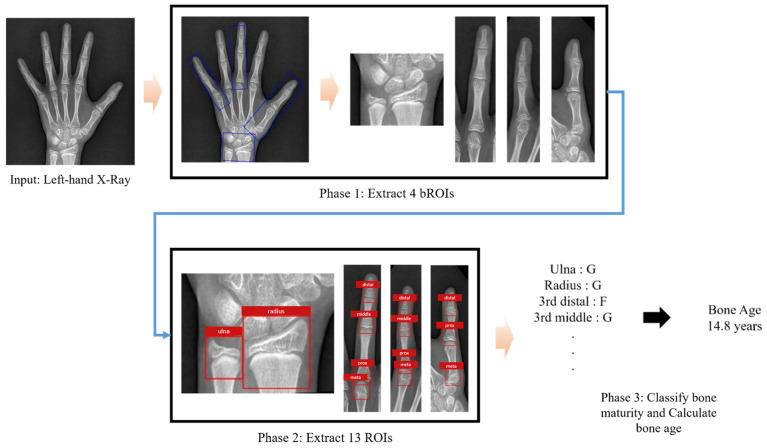
Workflow of automated bone age assessment using the TW3-based model. The model takes a left hand and wrist radiograph as input. It first detects and extracts bounding regions of interest (bROIs), followed by the identification of 13 Tanner–Whitehouse 3 (TW3)-specific regions of interest (ROIs). Each ROI is then individually assessed for skeletal maturity, and the results are aggregated to produce the final bone age estimate.

A commercialized TW3-based AI solution for automated bone age assessment was used in this study. The model utilized a combination of two convolutional neural networks, Rotated SSD and EfficientNet-B0. Rotated SSD was used for ROI extraction whereas EfficientNet-B0 was used for skeletal maturity assessment. Figure A1 illustrates the workflow of the model. The model was trained using 80% of 3344 hand and wrist radiographs of Korean population aged <18 years, labeled by two radiologists using the TW3 method, with the remaining images used for validation and testing. The training process was carried out over 100 epochs with a batch size of 64 and dynamic learning rate starting from 0.1. Batch normalization was utilized to mitigate internal covariate shift and accelerate the training process. For data augmentation, the model uses preprocessing steps including scaling and rotation normalization of bounding ROIs. Additionally, the model uses HSV (Hue, Saturation, Value) transformations and horizontal flipping. Horizontal flipping effectively doubles the dataset size by generating mirrored images, and helps the model generalize better across varying object orientations. The training was performed using 64 GB of RAM, an Intel^®^ Core™ i9-10980XE CPU @ 3.00 GHz (Intel Corporation, Santa Clara, CA, USA), and a 24-GB NVIDIA Titan RTX GPU (NVIDIA, Santa Clara, CA, USA). Further details can be found in the publication for the previous version of the model (HH-boneage.io; HealthHub Co., Ltd., Seoul, Republic of Korea), which was approved by the Korean Food and Drug Administration [27].

### 2.4. Outcomes

The primary outcome of this study was the comparison of bone age measurements between radiologists and the TW3-based AI model. Secondary outcomes included a comparison of bone age measurements by sex, a linear regression analysis of bone age measurements between the radiologists and TW3-based model, and an analysis of interobserver variability.

### 2.5. Statistical Analysis

The bone age measurements from the model and radiologists were compared using paired *t*-tests. Then, linear regression analysis was performed and the correlation coefficient (r) was calculated. The Durbin–Watson statistic was used to evaluate the independence of residuals, with values between 1.5 and 2.5 considered acceptable. To demonstrate the overall predictive performance of the model, RMSE and MAE were calculated. Bootstrapped 95% confidence intervals for RMSE and MAE were calculated using 1000 resampling iterations. Subsequently, a Bland–Altman analysis was conducted to evaluate the agreement between bone age measurements. The mean difference (bias) in bone age and 95% limits of agreement were reported. To further assess clinical equivalence, the proportion of cases exceeding an absolute difference of 0.6 years was calculated. Interobserver variability was assessed through linear regression analysis of bone age measurements between radiologists, and Cohen’s kappa coefficient (κ) for individual bone assessments in the TW3 method. All statistical analyses were conducted using IBM SPSS Statistics (version 27.0; IBM Corp., Armonk, NY 10504, USA), and figures were created using the R software (version 4.2.3. software, R Foundation for Statistical Computing, Vienna, Austria). A *p*-value < 0.05 was considered statistically significant.

## 3. Results

### 3.1. Characteristics of Study Participants

**Figure 1:**
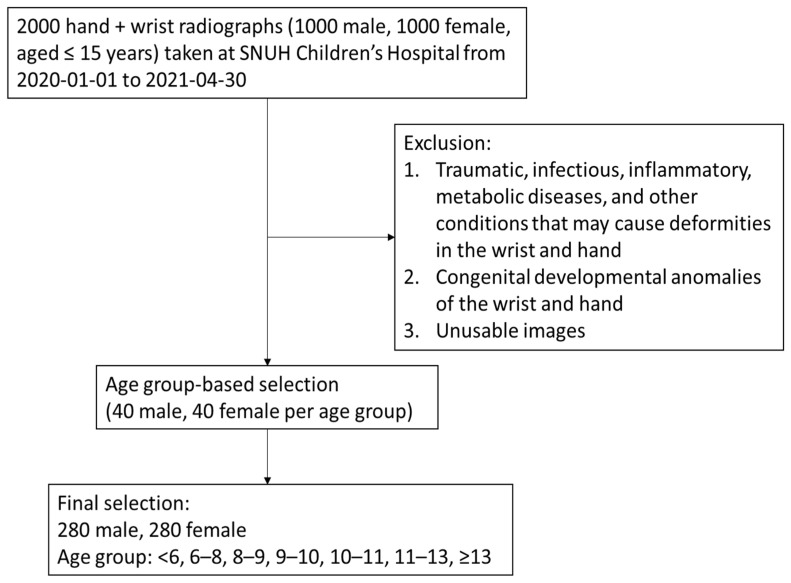
Study participant selection.

Figure 1 and Table 1 present the flow of participant selection and characteristics of the study population. After excluding pathological and unusable images, 560 subjects (280 females and 280 males) were included in the final analysis. The mean age of the participants was 9.43 ± 2.92 years. There were no significant differences in age between female and male groups.

### 3.2. Comparison of Bone Age Measurements Between the TW3-Based Model and Radiologists

Table 2 shows a comparison of the bone age measurements from the TW3-based AI model and consensus of board-certified pediatric radiologists. A total of 75.7% (424/560) of radiographs were interpreted by three radiologists whereas the remaining 24.3% (136/560) were interpreted by two radiologists. There was no significant difference between the bone age measurements from the TW3-based model and those from the radiologists. However, when analyzed by age group, the bone age predicted by the TW3-based model differed significantly from the radiologists’ measurements in participants <6 years old and those >13 years old (*p* < 0.001).

### 3.3. Linear Regression Analysis of Bone Age Measurements

**Figure 2:**
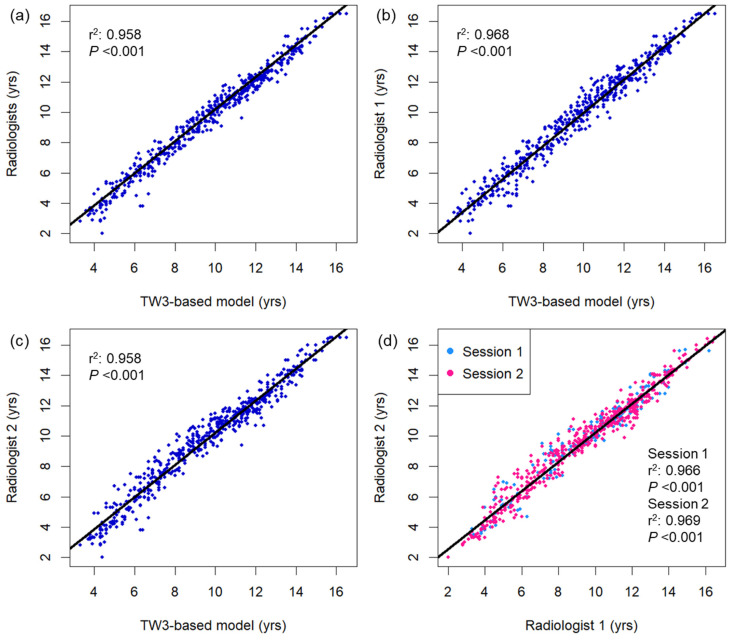
Bivariate scatterplots showing association between the bone age measurements using (**a**) the TW3-based model and radiologists, (**b**) TW3-based model and radiologist 1, (**c**) TW3-based model and radiologist 2, and (**d**) radiologists 1 and 2. The black lines represent the linear regression curves. DW = Durbin–Watson statistic.

**Figure A2:**
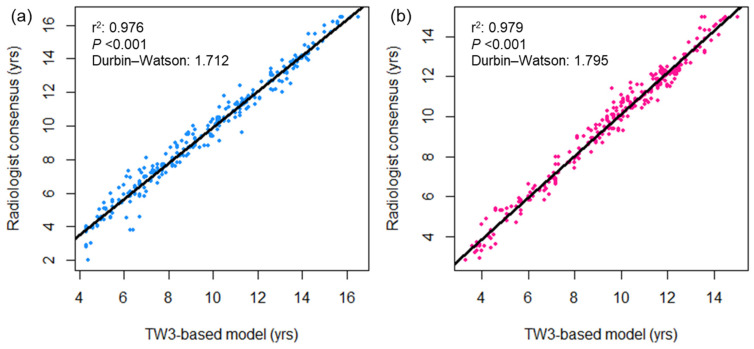
Bivariate scatterplots showing the association between bone age measurements by the TW3-based model and radiologists in the (**a**) male and (**b**) female subgroups. The black lines represent the linear regression curves. DW = Durbin−Watson statistic.

Figure 2 displays a comparison of bone age measured using the TW3-based model and pediatric radiologists. Strong linear relationships were observed between the TW3-based model prediction and radiologist consensus without significant autocorrelation in the residuals (r^2^ = 0.977, Durbin–Watson value = 1.635; Figure 2a), as well as individual radiologist measurements (r^2^ = 0.968, Durbin–Watson value = 1.603, radiologist 1; r^2^ = 0.958, Durbin–Watson value = 1.411, radiologist 2; Figure 2b,c). The two radiologists’ measurements also showed a strong linear correlation in both sessions of bone age evaluation, with some degree of correlation between the residuals (r^2^ = 0.966–0.969, Durbin–Watson value = 1.199–1.284; Figure 2d). Both male and female subgroups showed strong linear correlation between the model prediction and radiologist readings (male, r^2^ = 0.976, Durbin–Watson value = 1.712; female, r^2^ = 0.979, Durbin–Watson value = 1.795; Figure A2). Based on individual bone maturity scores, the two observers demonstrated similar consensus regarding intraobserver variability (κ = 0.991 for radiologist 1, and 0.986 for radiologist 2) and high interobserver variability (κ = 0.862–0.872; Table A1).

### 3.4. Bland–Altman Analysis of Bone Age Measurements

**Figure 3:**
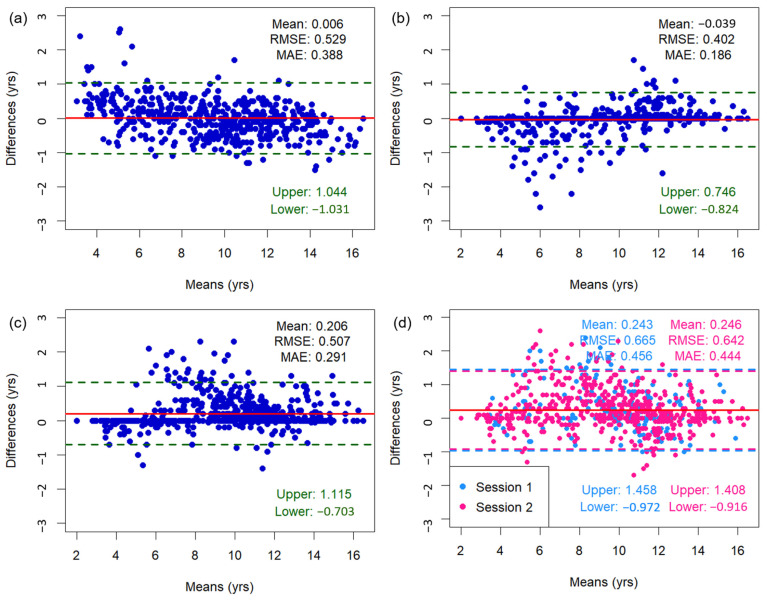
Bland–Altman plots illustrating the association between bone age measurements from (**a**) the TW3-based model and radiologists, (**b**) the TW3-based model and radiologist 1, (**c**) the TW3-based model and radiologist 2, and (**d**) radiologists 1 and 2. The upper and lower limits represent the 95% confidence intervals. The red line represents the mean difference. RMSE = root mean square error, MAE = mean absolute error.

**Figure A3:**
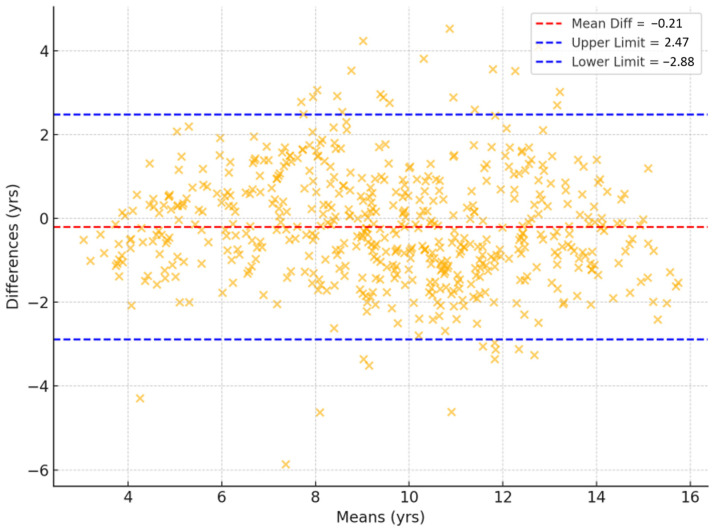
Bland–Altman plots illustrating the association between bone age prediction from the TW3-based model and chronological age. The upper and lower limits represent the 95% confidence intervals. RMSE = root mean square error, MAE = mean absolute error.

Figure 3 shows the Bland–Altman plots of bone age assessments, including mean differences, limits of agreement, as well as RMSE and MAE values. The mean difference between the TW3-based model predictions and the radiologist assessments was 0.006 years, with 95% limits of agreement ranging from −1.031 to 1.044 years. Outliers were concentrated among subjects <6 or >13 years old. When a sensitivity analysis was conducted excluding these age groups, the mean difference was 0.023 years and 95% limits of agreement ranged from −0.931 to 0.886 years. The overall RMSE and MAE of the TW3-based model were 0.529 and 0.388 years (95% CI for RMSE, −0.482–0.575 years; 95% CI for MAE, 0.361–0.417 years) (Figure 3a). The RMSE and MAE compared to those of specific radiologists were 0.402–0.507 and 0.186–0.291 years (Figure 3b,c). Between the radiologists, the RMSE and MAE were 0.642–0.665 and 0.444–0.456 years, respectively (Figure 3d). Additionally, the Bland–Altman plot comparing TW3-based model prediction with chronological age is presented in Figure A3.

Applying a tighter threshold for clinical applicability, the proportion of participants with absolute differences ≥ 0.6 years was 17.7%. However, the proportion was higher at 32.5% for participants < 6 years and 21.3% for participants > 13 years (Table A2).

## 4. Discussion

Reliable automated bone age assessment using deep learning is an area of active research. Herein, we evaluated the performance of a TW3-based AI model that can predict bone age, comparable to that of pediatric radiologists in the Korean pediatric population. Additionally, linear regression analysis confirmed a strong correlation between the model-estimated bone ages and those estimated by expert pediatric radiologists.

The reliability of bone age assessment depends largely on the methodology used for evaluation. When using the GP method, there is no standard method for weighing the differences between bones during assessment. Thus, the GP method is prone to larger inter- and intraobserver variability than the TW method [28]. King et al. measured that the average spread of bone age prediction was 0.96 years for the GP method, compared to 0.74 years for the TW2 method [11]. Our TW3-based model outperformed the GP method by achieving an RMSE of 0.529 years (6.35 months) and MAE of 0.388 years (4.66 months). Additionally, the estimation error in this study is smaller than the average error of recent AI systems (9.96 months) reported in a systematic review by Dallora et al., and is comparable to that of TW3-based AI systems (0.5 years) reported in prior studies involving Chinese and Caucasian populations [23,24,25,26]. Finally, the RMSE of our model was smaller than a generally accepted standard deviation of approximately 1 year for bone age measurement using the TW3 method, which likely reflects the inherent biological variability in skeletal maturation [9,29]. Therefore, the TW3-based model produced reliable and accurate predictions for bone age assessment.

The combination of the TW3 method with deep learning networks has great potential for accurate and efficient bone age measurements. Although the TW method is known for its accuracy and reproducibility, its clinical application is limited by its complexity and radiologist workload [28]. Deep learning models can alleviate these issues by automating the labor-intensive process of scoring multiple ROIs, including the radius, ulna, and short bones of the hand. The modular structure of the TW3 method also fits well with CNNs, which demonstrated excellent performance in feature extraction and classification. Evidence on improved efficiency is suggested by Booz et al., where AI-based bone age assessment (BoneXpert) effectively reduced the mean reading time by 87% [18]. The average inference time of our model was 32.6 s. Although we did not directly compare interpretation time in this study, further study focusing on the interpretation time could help validate the practicality of the model.

The deep learning architecture used in this study is based on Rotated SSD for ROI extraction and EfficientNet-B0 for skeletal maturity assessment. This design was chosen to improve upon the previous model by Shin et al. [21], which used Faster R-CNN and VGGNet-BA CNN. While Faster R-CNN’s two-stage detection process for ROI extraction is accurate, it is computationally intensive and slower in inference. In contrast, Rotated SSD’s one-stage mechanism offers faster processing and rotation-invariant capabilities, making it more applicable for images with varied orientations. Additionally, replacing VGGNet-BA CNN with EfficientNet-B0 provides comparable or superior performance with fewer parameters, reducing computational load and improving efficiency, which is essential in clinical settings. Likewise, in contrast to state-of-the-art architectures like Vision Transformers [30,31], which produce 0.3–0.6 years of error and require substantial computational power, this model can be operated on standard CPU hardware without GPU acceleration and offers better practicality for physicians without incurring additional infrastructure costs.

There were some discrepancies in the bone age predicted by the model and radiologists in participants aged <6 and >13 years. Similar deviations in bone age prediction for younger and older children were reported by Kim et al., who examined the performance of the GP-based model and its modified version in a Korean population [32]. This may be contributed to the ethnic differences in skeletal maturation, as both the GP and TW3 methods were originally developed for Caucasian populations [5,9]. In Asian populations, skeletal maturation starts later and ends earlier than in Caucasians, resulting in delayed bone age in children and advanced bone age in adolescents [33]. Experienced local radiologists consider ethnic variations during manual assessments; however, AI models trained using Caucasian-population-based references could produce systematic errors in age groups with significant ethnic differences. To address this issue, further studies incorporating ethnicity-based references may enhance the robustness of the AI systems across a broader age range.

AI-based bone age measurement holds significant clinical implications, especially in pediatric endocrinology. Whereas traditional bone age assessment by manual evaluation of radiologists can be time-consuming and resource-dependent, AI offers the potential for clinicians to easily access an automatically generated bone age estimate which can be compared to the patient’s chronological age. A significant disparity between the two values would prompt further diagnostic tests to identify pathological conditions that may contribute to accelerated or delayed growth. The facilitated evaluation could contribute to improved patient outcomes and reduced healthcare costs. However, the clinical integration of AI will require careful consideration, as the clinicians must be able to trust the model’s performance and understand its limitations.

This study has several limitations. First, the participants were recruited from a single institution, which may have introduced selection bias. Second, the sample size was relatively small, and larger prospective studies are required to validate our findings. Third, the study was based on the Korean population, so generalizability may be limited when applying it to different ethnic groups. Lastly, the model was not tested on radiographs with obvious bony destruction and congenital anomalies. In such cases, manual bone age assessments would still be necessary for clinical decision making.

## 5. Conclusions

The TW3-based AI model provides accurate and fully automated bone age prediction, potentially reducing the burden on radiologists and facilitating clinical decision-making.

## Figures and Tables

**Table 1:** Characteristics of study participants.

	Female	Male	*p*-Value
*N*	280	280	
Age (mean ± SD, yrs)			
Overall	9.42 ± 2.86	9.45 ± 2.98	0.895
<6	4.63 ± 0.98	4.51 ± 0.88	0.554
6–8	7.11 ± 0.59	6.98 ± 0.55	0.301
8–9	8.60 ± 0.30	8.52 ± 0.34	0.258
9–10	9.47 ± 0.28	9.58 ± 0.31	0.113
10–11	10.45 ± 0.27	10.49 ± 0.32	0.544
11–13	11.90 ± 0.67	12.06 ± 0.55	0.240
>13	13.76 ± 0.64	14.02 ± 0.62	0.070

SD, standard deviation.

**Table 2:** Bone age measurements from the Tanner–Whitehouse-based model compared to radiologists (*n* = 560).

Age (yrs)	Mean ± SD ^1^	TW3-Based Model	Radiologists ^2^	*p*-Value ^3^
Overall	9.43 ± 2.93	9.64 ± 3.05	9.64 ± 3.26	0.874
<6	4.57 ± 0.94	5.22 ± 1.27	4.73 ± 1.44	<0.001
6–8	7.04 ± 0.58	7.09 ± 1.41	7.08 ± 1.51	0.737
8–9	8.56 ± 0.33	8.70 ± 1.36	8.79 ± 1.59	0.093
9–10	9.52 ± 0.30	9.76 ± 1.47	9.80 ± 1.54	0.301
10–11	10.47 ± 0.30	10.94 ± 1.36	10.91 ± 1.46	0.584
11–13	11.98 ± 0.62	12.15 ± 1.38	12.15 ± 1.50	0.976
>13	13.89 ± 0.65	13.65 ± 1.63	14.00 ± 1.63	<0.001

^1^ Mean value and standard deviation of chronological age were presented. ^2^ Radiologists’ measurements were set by consensus of three board-certified pediatric radiologists. ^3^ *p*-Values were obtained by comparing the bone age measurement from the TW3-based model and radiologists.

## Data Availability

The data presented in this study are available on request from the corresponding author.

## Appendix A

**Table A1:** Intraobserver and interobserver variability (κ) of maturity measurements for specific ROIs in the Tanner–Whitehouse 3 method.

ROI ^1^	Radiologist 1	Radiologist 2	Interobserver
Ulna	0.993	0.991	0.927–0.936
Radius	0.986	0.975	0.820–0.845
3rd Distal Phalanx	0.995	0.994	0.890–0.896
3rd Mid Phalanx	0.992	0.988	0.878–0.882
3rd Proximal Phalanx	0.983	0.991	0.920–0.924
3rd Metacarpal Bone	0.998	0.948	0.763–0.792
5th Distal Phalanx	0.991	0.997	0.861–0.871
5th Mid Phalanx	0.980	0.994	0.845–0.852
5th Proximal Phalanx	0.992	0.981	0.863–0.865
5th Metacarpal Bone	0.992	0.963	0.725–0.752
1st Distal Phalanx	0.991	0.990	0.828–0.839
1st Proximal Phalanx	0.994	0.995	0.881–0.885
1st Metacarpal Bone	0.992	0.991	0.861–0.872
Total	0.991	0.986	0.862–0.872

^1^ ROI = Region of interest. ROI was used for scoring skeletal maturity.

**Table A2:** Proportion of subjects with bone age estimation absolute error below 0.6 years.

Age (yrs)	Female	Male	Total
<6	8 (20.0%)	18 (45.0%)	26 (32.5%)
6–8	4 (10.0%)	7 (17.5%)	11 (13.8%)
8–9	11 (27.5%)	6 (15.0%)	17 (21.3%)
9–10	5 (12.5%)	6 (15.0%)	11 (13.8%)
10–11	4 (10.0%)	10 (25.0%)	14 (17.5%)
11–13	2 (5.0%)	1 (2.5%)	3 (3.8%)
>13	8 (20.0%)	9 (22.5%)	17 (21.3%)
Total	42 (15.0%)	57 (20.4%)	99 (17.7%)
